# An exploration of testing genetic associations using goodness-of-fit statistics based on deep ReLU neural networks

**DOI:** 10.3389/fsysb.2024.1460369

**Published:** 2024-11-18

**Authors:** Xiaoxi Shen, Xiaoming Wang

**Affiliations:** Department of Mathematics, Texas State University, San Marcos, TX, United States

**Keywords:** deep neural networks, goodness-of-fit test, asymptotic normality, sample splitting, genetic association

## Abstract

As a driving force of the fourth industrial revolution, deep neural networks are now widely used in various areas of science and technology. Despite the success of deep neural networks in making accurate predictions, their interpretability remains a mystery to researchers. From a statistical point of view, how to conduct statistical inference (e.g., hypothesis testing) based on deep neural networks is still unknown. In this paper, goodness-of-fit statistics are proposed based on commonly used ReLU neural networks, and their potential to test significant input features is explored. A simulation study demonstrates that the proposed test statistic has higher power compared to the commonly used t-test in linear regression when the underlying signal is nonlinear, while controlling the type I error at the desired level. The testing procedure is also applied to gene expression data from the Alzheimer’s Disease Neuroimaging Initiative (ADNI).

## Introduction

Since the creation of backpropagation, neural networks have regained their popularity, and deep neural networks are now the fundamental building blocks of sophisticated artificial intelligence. For instance, in computer vision, convolutional neural networks (CNNs) (LeCun, 1989) are commonly used for object detection, while recurrent neural networks (RNNs) (Rumelhart et al., 1988), or more recently, transformers (Vaswani et al., 2017) play vital roles in natural language processing.

One of the main reasons for the superior performance of deep learning models is that neural networks are universal approximators. In fact, in the early 1990s, various research established the universal approximation property for shallow neural networks, as well as their derivatives with squashing activation functions—functions that are monotonically increasing and approach 0 and 1 when the variable tends to negative and positive infinity, respectively (Cybenko, 1989; Hornik et al., 1989; Pinkus, 1999) showed that any neural network has the universal approximation property as long as the activation function is not a polynomial. Recently, similar results have also been established for deep neural networks with the Rectified Linear Unit (ReLU) activation function (Nair and Hinton, 2010). Another important characteristic of shallow neural networks is that the approximation rate to certain smooth functions is independent of the dimensionality of the input features (Barron, 1993), making neural networks a great candidate to avoid curse of dimensionality. For example (Shen et al., 2023; Braun et al., 2024), have shown that the rate of convergence of shallow neural networks is independent of the input dimension when the underlying function resides in the Barron space.

Such nice approximation properties provide deep neural networks with great potential for modeling complex genotype-phenotype relationships, and a lot of research has been done in this direction. For instance, a deep learning method known as DANN (Quang et al., 2014) was proposed to make predictions on the deleteriousness of genetic variants. In terms of predicting effects of the non-coding regions, DanQ (Quang and Xie, 2016) integrated CNNs and Bidirectional Long Short-Term Memory networks to capture different aspects of DNA sequences and outperformed other similar methods in various metrics. More recently (Zhou et al., 2023), used deep neural networks to model Alzheimer’s disease (AD) polygenic risk and the deep learning methods outperform traditional methods such as weighted polygenic risk score model and LASSO (Tibshirani, 1996).

Despite empirical and theoretical evidence on the powerful prediction performance of deep neural networks, an overlooked problem in deep learning is the interpretability of these models. From a statistical perspective, the interpretability of deep learning models can be improved if we know how to conduct statistical inference using deep neural networks. In recent years, several works have been done in this direction. For example (Horel and Giesecke, 2019), proposed a significant test based on shallow neural network using empirical process theory. However, the asymptotic distribution of the test statistic is hard to compute. Recently, Shen et al. (2021) and Shen et al. (2022) proposed two testing procedures for shallow neural networks with sigmoid activation function. Both of these testing procedures are easier to implement and have better performance compared to *t*-test or *F* test in linear regression. Dai et al. (2024) also proposed a black box testing procedure to test conditional independence between features and response. Below we would like to point out several challenges one needs to conquer in order to develop hypotheses testing based on deep learning models:

1. Classical statistical hypothesis testing techniques in parametric models are difficult to apply in DNNs. One reason is that the parameters (weights and biases) are unidentifiable in general (Fukumizu, 2003), making them hard to interpret. For example, in linear regression, testing the significance of a covariate is equivalent to testing the coefficient attached to it is equal to 0 or not. However, in a DNN, there are many ways to make the covariate vanish in the model. As an example one can let all the weights directly attached to an input feature be 0 or one can also let all the weights for each hidden-to-output unit to be 0.
2. The number of tuning parameters to train a DNN is large. There is no general guideline on how to choose the number of layers and the number of hidden units in each layer to achieve desirable performance in a DNN. Additionally, in the training process, how to wisely select the learning rate and the number of iterations needed is also unclear. Without carefully choosing these tuning parameters, it is likely that the trained DNN will overfit the data. Although overfitting might be acceptable for prediction, it generally needs to be avoided when conducting statistical hypothesis testing.
3. There is lack of theoretical guarantees to ensure the performance of DNNs as tools in genetic association studies. Current theories on DNNs mainly focus on evaluating the generalization errors of DNNs. Many results available are based on the assumption of high-dimensional regime, where the sample size and the number of features are of the same order, or in the polynomial regime, where the sample size grows polynomially as the number of features (Mei et al., 2022; Mei and Montanari, 2022). These conditions are easily satisfied in tasks like image classification, where one can use the data augmentation strategy to manually generate new samples. In genetic studies, however, researchers usually face a limited sample size but a huge number of genetic variants, making those results less attractive in genetic studies.

In this paper, we proposed a goodness-of-fit test based on deep ReLU neural networks, extending the work of (Shen et al., 2021). The rest of the paper is organized as follows: Section 2 provides a brief introduction to deep neural networks, followed by the proposed goodness-of-fit test. Results from simulation studies and real data analyses are presented in Section 3, and conclusions are drawn in Section 4.

## Methods

### Deep neural networks (DNNs)

**FIGURE 1:**
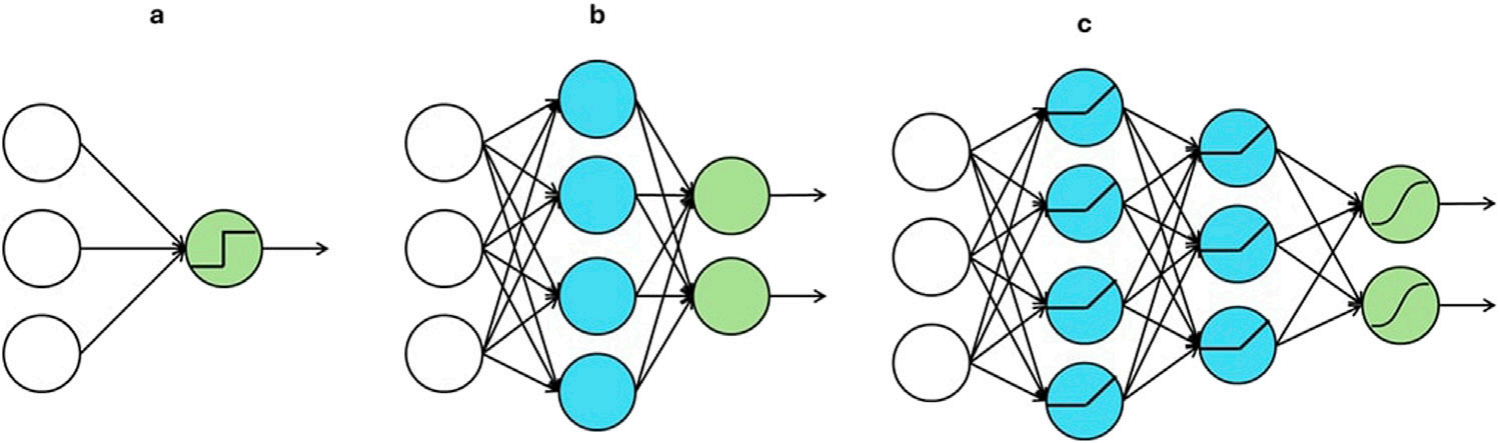
Architectures of **(A)** a perceptron, **(B)** a shallow neural network and **(C)** a deep neural network.

A perceptron (Rosenblatt, 1958) originated from mimicking the functionality of a neuron in the human brain. As shown in Figure 1A, the green node is the only computation unit in a perceptron, and it outputs a nonlinear transformation of the linear combination of input units. Such a transformation in a computation unit is often called an activation function. By stacking multiple perceptrons together, a shallow neural network, shown in Figure 1B, is obtained. The blue computation nodes in the middle are known as the hidden units. Each of them computes a nonlinear activation of a linear combination of the nodes in the input layer. The green nodes are known as output units, and each of them applies a linear or nonlinear activation to a linear combination of the outputs from the hidden units. When the number of hidden layers is more than one, as shown in Figure 1C, a deep neural network is obtained.

Throughout the remainder of the paper, we consider deep neural networks with only one output unit and linear activation is applied to the output unit. In particular, the output of a deep neural network with *L* hidden layer can be represented as

$$f\left(x\right)=W_{L+1}\sigma \left(W_{L}\sigma \left(\cdots W_{2}\sigma \left(W_{1}x\right)\right)\right),$$ (1)

where $W_{l}$ is an $n_{l}\times n_{l-1}$ matrix containing the weights between the (*L*-1)th layer and the *l*th layer. Here $n_{l}$ is the number of nodes in the *l*th layer. By convention, the 0^th^ layer represents the input layer, while the (*l*+1)th layer represents the output layer and therefore, $n_{0}=p$ and $n_{L+1}=1$ by our model assumption. σ:R→R is a nonlinear activation function and in this paper, we considered one of the most used nonlinear activation functions, the Rectified Linear Unit (ReLU) activation function (Nair and Hinton, 2010). That is, $\sigma \left(x\right)=\max\left\{x,0\right\}$ . In (1), when σ is applied to a matrix or a vector, it is considered as an elementwise operation.

### Goodness-of-fit test based on DNNs

We consider the following nonparametric regression model:

$$Y_{i}=f_{0}\left(X_{i}\right)+\varepsilon_{i},i=1,\ldots ,n$$

where $\left(X_{i},Y_{i}\right),i=1,\ldots ,n$ are i.i.d pairs of data points with $X_{i}={\left[X_{i1},\ldots ,X_{ip}\right]}^{T}\in R^{p}$ being the vector of covariates for the *i*th individual and $Y_{i}$ being the response for the *i*th individual. $\varepsilon_{1},\ldots ,\varepsilon_{n}$ are i.i.d. random errors with mean 0 and variance $\sigma^{2}$ . Moreover, $f_{0}$ is an underlying function to be estimated using deep neural networks through minimizing the squared error loss:

$${\hat{f}}_{n}=argmin_{f\in F_{DNN}}\frac{1}{n}\sum_{i=1}^{n}{\left(Y_{i}-f\left(X_{i}\right)\right)}^{2},$$

where $F_{DNN}$ is the class of deep neural networks of the form Equation 1, that is,

$$F_{DNN}=\left\{f\left(x\right)=W_{L+1}\sigma \left(W_{L}\sigma \left(\cdots W_{2}\sigma \left(W_{1}x\right)\right)\right):{\left\|f\right\|}_{\infty }\leq M\right\}.$$

In addition, we assume that $X_{i}$ come from a continuous distribution, $Y_{i}\in \left[-M,M\right]$ for some *M* > 0 and the underlying function is bounded, that is ${\left\|f_{0}\right\|}_{\infty }\leq M$ . These assumptions are required to provide an upper bound for ${\left\|{\hat{f}}_{n}-f_{0}\right\|}_{L^{2}}$ as demonstrated in (Farrell et al., 2021).

Our goal is to develop a statistical hypothesis testing procedure to test whether certain covariates should be included in the model or not based on the deep neural network estimator ${\hat{f}}_{n}$ . In other words, for $S\subset \left\{1,\ldots ,p\right\}$ , a subset of indices of covariates, the null hypothesis is $H_{0}:X_{j},j\in S$ are not significant. To gain some insights of the testing procedure, recall that in multiple linear regression, testing the significance of a predictor is equivalent to testing whether its coefficient is zero or not. This is the well-known t-test procedure. However, due to the unidentifiability of neural network parameters, such a method cannot be easily applied to neural networks. On the other hand, such a *t*-test is equivalent to an *F* test by comparing the mean squared error under the full model where the predictor is involved and the reduced model where the predictor is excluded from the model. Our goodness-of-fit test for deep neural networks is constructed based on such an idea.

Following (Shen et al., 2021), we proposed to use a goodness-of-fit (GoF) type statistic for genetic association studies using DNNs. Here are the steps to construct the GoF test statistic.

1. Randomly partitioned the dataset into two parts. Denote 0<γ≤0.5 to be the proportion of the first part among the total *n* data points. Also let m=⌊γn⌋ be the number of data points in the first part so that n−m is the number of data points in the second part. For simplicity, we denote $\left(X_{1},Y_{1}\right),\ldots ,\left(X_{m},Y_{m}\right)$ to be the first part of the data and $\left(X_{m+1},Y_{m+1}\right),\ldots ,\left(X_{n},Y_{n}\right)$ to be the second part of the data.
2. Use the first part is used to fit the data under the null hypothesis $H_{0}$ and this is done by training a deep neural network whose input layer only involves the covariates $X_{j},j\notin S$ . The second part is used to fit the data under the alternative hypothesis which is done by fitting a deep neural network using all the covariates. The mean squares errors of these two model fittings are given by

$$T_{0}=\frac{1}{m}\sum_{i=1}^{m}{\left(Y_{i}-{\hat{f}}_{H_{0}}\left(X_{i}\right)\right)}^{2},$$

$$T_{1}=\frac{1}{n-m}\sum_{i=m+1}^{n}{\left(Y_{i}-{\hat{f}}_{H_{1}}\left(X_{i}\right)\right)}^{2}.$$

3. The asymptotic distribution of $T_{0}$ and $T_{1}$ can be obtained in a similar fashion as of (Shen et al., 2021). Combining Lemma 3 in (Shen et al., 2021) and Theorem 2 in (Farrell et al., 2021), it follows that under the null hypothesis *H*_0_, both $T_{0}$ and $T_{1}$ are asymptotically standard normally distributed when $B_{n}L_{n}\log B_{n}\log n=o\left(n\right)$ where $B_{n}$ is the number of parameters in the DNN and $L_{n}$ is the number of hidden layers in the DNN. Therefore,

$${\left[\left(\frac{1}{m}+\frac{1}{n-m}\right)\kappa \right]}^{-\frac{1}{2}}\left(T_{0}-T_{1}\right)\overset{d}{\to }N\left(0,1\right),$$

where $\kappa =E\left(\varepsilon^{4}\right)$ is the fourth moment of the random error provided that $B_{n}L_{n}\log B_{n}\log n=o\left(n\right)$ .

4. The GoF test statistic can be obtained by replacing κ by a consistent estimator:

$$T={\left[\left(\frac{1}{m}+\frac{1}{n-m}\right)\;{\hat{\kappa }}_{n}\right]}^{-\frac{1}{2}}\left(T_{0}-T_{1}\right),$$

As mentioned in (Yatchew, 1992), a possible choice for ${\hat{\kappa }}_{n}$ is

$${\hat{\kappa }}_{n}=\frac{1}{n}\sum_{i=1}^{n}{\left(Y_{i}-{\hat{f}}_{H_{0}}\left(X_{i}\right)\right)}^{4}-{\left(\frac{1}{n}\sum_{i=1}^{n}{\left(Y_{i}-{\hat{f}}_{H_{0}}\left(X_{i}\right)\right)}^{2}\right)}^{2}.$$

5. The *p*-value of the test is then calculated the same way as in a two-sided Z-test. In other words, $p=P\left(\left|T\right|>\left|t\right|\right)$ , where *t* is the observed test statistic.

### Network structures

A sufficient condition, as has been mentioned above, to ensure asymptotic normality is $B_{n}L_{n}\log B_{n}\log n=o\left(n\right)$ . In fact, this condition provides some guidance on how to choose the network structure. Since $B_{n}$ is the number of parameters in a DNN, $B_{n}≍n^{{*}^{2}}L_{n}$ , where $n^{*}=\max\left\{n_{1},\ldots ,n_{L_{n}}\right\}$ . Therefore, $B_{n}L_{n}\log B_{n}\log n≍n^{{*}^{2}}L_{n}^{2}\log\left(n^{*}L_{n}\right)\log n$ . Now we consider the following scenarios:

• If $L_{n}=O\left(1\right)$ , such as a shallow ReLU neural network, then the sufficient condition is equivalent to $n^{{*}^{2}}\log n^{*}\log n=o\left(n\right)$ . In this case, one can choose $n^{*}=O\left(n^{\frac{1}{2}-\alpha }\right)$ for some $0<\alpha <\frac{1}{2}$ .
• If $n^{*}=O\left(1\right)$ , i.e., each hidden layer has a bounded number of hidden units, then the sufficient condition is equivalent to $L_{n}^{2}\log L_{n}\log n=o\left(n\right)$ . In this case, one can choose $L_{n}=O\left(n^{\frac{1-\alpha }{2}}\right)$ for some 0<α<1 .
• If both $n^{*}$ and $L_{n}$ can increase with the sample size, then one can choose $n^{*}=O\left(n^{\alpha }\right)$ and $L_{n}=O\left(n^{\beta }\right)$ as long as α and β satisfy $0<\alpha +\beta <\frac{1}{2}$ .

## Results

### Simulation 1

In this section, we conducted a simulation study to evaluate our proposed test’s type I error and power. Since in genetic studies, linear models are the most used method to detect genetic associations, we compared our proposed test with the t-test in linear regression. Specifically, we generated the response variable via the following equation:

$$Y_{i}=f_{0}\left(X_{i1}\right)+\varepsilon_{i},i=1,\ldots ,n,$$

where $X_{i}={\left[X_{i0},X_{i1}\right]}^{T},i=1,\ldots ,n$ are i.i.d. random vectors sampled from a uniform distribution on the square ${\left[-1,1\right]}^{2}$ . $\varepsilon_{i},i=1,\ldots ,n$ are i.i.d. random variables sampled from a normal distribution $N\left(0,0.5^{2}\right)$ . In the simulation, we consider two different functions $f_{0}$ . One is the quadratic function $f_{0}\left(x\right)=x^{2}$ and the other one is a trigonometric function $f_{0}\left(x\right)=\cos\left(2\pi x\right).$

Since the first component does not involve in the simulation equation, it was used to evaluate the performance of the type I error of the proposed test. The null hypothesis to be tested is $H_{0}:X_{0}$ is not significant, or equivalently, the index set for this null hypothesis is $S=\left\{0\right\}$ . The second component in $X_{i}$ was involved in generating the response, it was therefore to be used to evaluate the power of the proposed test. In this case, the null hypothesis to be tested is $H_{0}:X_{1}$ is not significant, or equivalently, the index set for this null hypothesis is $S=\left\{1\right\}$ . To test significance of each component, we applied the testing procedure as mentioned above. We started by partitioning the data set into two parts with ratio γ=0.1 and γ=0.5 . Then the majority of the data was used to train a shallow or a deep ReLU neural network under the alternative hypothesis while the minority of the data was used to calculate the mean squared error under the null hypothesis. When we trained the neural networks, the following three network structures were used:

• A shallow ReLU neural network with the number of hidden units being $\left\lfloor n^{1/3}\right\rfloor$ .
• A deep ReLU neural network with the number of hidden layer being $\left\lfloor n^{1/3}\right\rfloor$ and each hidden layer has 18 hidden units.
• A deep ReLU neural network with $\left\lfloor n^{1/4}\right\rfloor$ hidden layers and each hidden layer has $\left\lfloor n^{1/4}\right\rfloor$ hidden units.

All the three network structures used here meet the requirement as mentioned in section 2.3. In the simulation, we considered sample sizes being 200, 500, 1,000 and 2000. The stochastic gradient descent algorithm was applied, and the batch size was determined so that 20 batches were used for each sample size. 200 epochs were used to run the stochastic gradient descent. To further alleviate the possible overfitting, we applied dropout to each hidden unit in the network with a dropout rate being 0.05. To obtain the empirical type I error and the empirical power, 1,000 Monte Carlo replications were conducted. Tables 1, 2 below summarize the simulation results.

**TABLE 1:** Comparisons between linear model and goodness-of-fit test based on ReLU neural networks under quadratic signal.

		γ=0.1				γ=0.5			
Sample size		200	500	1,000	2,000	200	500	1,000	2,000
Type I Error	Linear Model	0.047	0.047	0.055	0.048	0.041	0.041	0.038	0.054
	Shallow ReLU NN	0.028	0.053	0.050	0.053	0.102	0.066	0.056	0.053
	Deep ReLU NN 1	0.030	0.054	0.049	0.052	0.108	0.066	0.053	0.050
	Deep ReLU NN 2	0.046	0.048	0.039	0.042	0.088	0.061	0.055	0.051
Power	Linear Model	0.058	0.071	0.068	0.076	0.073	0.068	0.058	0.063
	Shallow ReLU NN	0.152	0.367	0.580	0.858	0.484	0.736	0.955	1.000
	Deep ReLU NN 1	0.098	0.295	0.543	0.787	0.594	0.774	0.952	0.998
	Deep ReLU NN 2	0.056	0.176	0.448	0.738	0.273	0.513	0.830	0.944

**TABLE 2:** Comparisons between linear model and goodness-of-fit test based on ReLU neural networks under cosine signal.

		γ=0.1				γ=0.5			
Sample size		200	500	1,000	2,000	200	500	1,000	2,000
Type I Error	Linear Model	0.063	0.046	0.062	0.051	0.055	0.048	0.049	0.060
	Shallow ReLU NN	0.057	0.050	0.056	0.063	0.072	0.079	0.056	0.050
	Deep ReLU NN 1	0.054	0.048	0.056	0.059	0.081	0.075	0.048	0.050
	Deep ReLU NN 2	0.039	0.061	0.040	0.052	0.064	0.076	0.048	0.052
Power	Linear Model	0.051	0.058	0.061	0.055	0.062	0.050	0.043	0.068
	Shallow ReLU NN	0.106	0.483	0.876	0.952	0.551	0.858	0.966	0.996
	Deep ReLU NN 1	0.228	0.295	0.413	0.425	0.970	0.982	0.981	0.922
	Deep ReLU NN 2	0.042	0.083	0.262	0.622	0.218	0.541	0.789	0.911

Based on Tables 1, 2, it can be easily seen that linear models and the proposed GoF test can control the empirical type I error very well at level 0.05, except that the proposed GoF test is slightly conservative when the sample size is small for the quadratic signal for the split-ratio γ=0.1 , while the empirical type I error rate of the GoF test is slightly inflated for small sample size when the split ratio γ=0.5 . The empirical powers of proposed GoF test based on ReLU neural networks are consistently much higher compared to the *t*-test in linear model, which suggests that the proposed GoF test can outperform the *t*-test in linear model when the underlying signal is nonlinear. On the other hand, it is worth noting that when γ=0.1, shallow ReLU neural networks achieve higher empirical power than deep ReLU neural networks in both cases, especially when the sample size is relatively large. On the contrary, when the underlying function is the cosine function and the sample size is 200, deep ReLU neural networks have higher power compared to the shallow ones. Similar situations can also be seen for γ=0.5 , but for the cosine signal, deep neural networks with structure 1 (growing number of hidden layers and fixed number of hidden units in each layer) achieve higher power compared to shallow neural networks. Therefore, we believe that these observations suggest that the rule of parsimony still applies in ReLU neural networks.

### Simulation 2

In many situations, a response variable can be related to multiple causal variables. In this simulation, we investigated the performance of the proposed method under such a scenario. In particular, the response variable in this simulation was generated based on the following equation:

$$Y_{i}=\left|X_{1i}\right|+2X_{2i}^{2}+\cos\left(2\pi X_{3i}\right)+\epsilon_{i},$$

where all the covariates $X_{0i},X_{1i},X_{2i},X_{3i}$ are i.i.d. random variables from Uniform[-1,1]. The random error term is sampled from $N\left(0,0.5^{2}\right)$ . Similar to Simulation 1, the variable $X_{0}$ is not involved in the underlying function, so it was used to check type I error of the test, and the other three variables were used to evaluate the power of the test.

In this scenario, the hypotheses of interest are $H_{0}:X_{j}$ is not significant for j∈S with $S=\left\{0\right\}$ for type I error and $S=\left\{1\right\},\left\{2\right\},\left\{3\right\}$ respectively for the three variables used to evaluate power. We used the same deep neural network structures and the same choices of tuning parameters as we did in Simulation 1. Table 3 summarize the empirical type I error rates and the empirical power of the proposed method, linear model, and the black-box test under the sample sizes 200, 500, 1,000, and 2,000.

**TABLE 3:** Comparisons between linear model and goodness-of-fit test based on ReLU neural networks under multiple causal variables.

		γ=0.1				γ=0.5			
Sample size		200	500	1,000	2,000	200	500	1,000	2,000
Type I Error ( $X_{0}$ )	Linear Model	0.058	0.046	0.044	0.043	0.052	0.047	0.056	0.048
	Shallow ReLU NN	0.046	0.043	0.044	0.064	0.076	0.064	0.048	0.054
	Deep ReLU NN 1	0.044	0.044	0.045	0.065	0.071	0.061	0.046	0.055
	Deep ReLU NN 2	0.047	0.043	0.042	0.063	0.063	0.064	0.046	0.054
Power ( $X_{1}$ )	Linear Model	0.066	0.061	0.056	0.042	0.040	0.045	0.049	0.041
	Shallow ReLU NN	0.049	0.064	0.108	0.127	0.128	0.134	0.172	0.287
	Deep ReLU NN 1	0.050	0.068	0.070	0.078	0.130	0.131	0.136	0.181
	Deep ReLU NN 2	0.048	0.055	0.058	0.074	0.084	0.072	0.075	0.107
Power ( $X_{2}$ )	Linear Model	0.081	0.075	0.065	0.062	0.074	0.065	0.070	0.087
	Shallow ReLU NN	0.057	0.387	0.710	0.967	0.533	0.859	0.974	0.998
	Deep ReLU NN 1	0.076	0.106	0.119	0.146	0.514	0.777	0.912	0.952
	Deep ReLU NN 2	0.051	0.057	0.072	0.321	0.170	0.361	0.647	0.834
Power ( $X_{3}$ )	Linear Model	0.045	0.055	0.065	0.059	0.040	0.050	0.054	0.064
	Shallow ReLU NN	0.046	0.082	0.373	0.568	0.163	0.228	0.273	0.314
	Deep ReLU NN 1	0.054	0.093	0.203	0.263	0.404	0.633	0.749	0.666
	Deep ReLU NN 2	0.050	0.042	0.055	0.119	0.077	0.111	0.171	0.309

As we can see from Table 3, both linear model t-test and the proposed GoF test can control the type I error rate very well. Similar to what we saw from Simulation 1, even the underlying function contains multiple causal variables, the proposed GoF test can still detect the significance of the variables having nonlinear associations with the response variable.

### Real data analyses

Alzheimer’s disease (AD) is one of the most common neurodegenerative diseases with a substantial genetic component (Karch et al., 2014; Sims et al., 2020). Therefore, it is of great importance to have an efficient method to screen the genetic components that are associated with AD pathogenesis so that early treatments can be applied for disease management (Zissimopoulos et al., 2015). To investigate the performance of our proposed GoF test in identifying AD-related genes, we applied our proposed method to the gene expression data from Alzheimer’s Disease Neuroimaging Initiative (ADNI).

The hippocampus region plays a vital role in memory (Mu and Gage, 2011) and the shrinkage of hippocampus volume is an early symptom of AD (Schuff et al., 2009). Therefore, we chose the hippocampus volume as the phenotype in the real data analysis. After removing individuals with missing values for hippocampus volume and merging data from individuals having both gene expression information and hippocampus volume, a total of 464 individuals and 15,837 gene expressions were obtained. We then regressed the scaled hippocampus volume onto some important predictors including age, gender and education status. The residual obtained will be used as the response variable to train ReLU neural networks. The network structures and hyperparameters in the ReLU neural networks used in the real data analysis were the same as in the simulation studies. Table 4 summarizes the top 10 significant genes selected from *t*-test in linear model and the GoF tests based on ReLU neural networks.

**TABLE 4:** Top 10 significant genes selected from *t*-test in linear model and the GoF tests based on different ReLU neural network structures.

Linear model	Shallow ReLU neural network	Deep ReLU neural network 1	Deep ReLU neural network 2
*SNRNP40*	*GRM2*	*GRM2*	*GRM2*
*PPIH*	*DGCR6*	*DGCR6*	*DGCR6*
*GPR85*	*GPRC5D*	*BRCA2*	*NDRG1*
*DNAJB1*	*SMARCB1*	*KIF1C*	*GPRC5D*
*WDR70*	*NDRG1*	*NDRG1*	*KIF1C*
*CYP4F2*	*KIF1C*	*GPRC5D*	*KLF13*
*NOD2*	*NUDT22*	*NUDT22*	*COX20*
*MEGF9*	*BRCA2*	*COX20*	*NUDT22*
*CTBP1-AS2*	*COX20*	*SMARCB1*	*OR4A5*
*PHYKPL*	*REG1A*	*STAG3L4*	*STAG3L4*

As can be seen from Table 4, the significant genes selected from the GoF test do not overlap with the ones selected from the linear models, and different network structures picked out similar genes. On the other hand, in (Shen et al., 2022), the top 10 significant genes selected using a testing procedure based on shallow sigmoid neural networks have large overlap with the ones selected from the linear model. This indicates that ReLU neural networks may be able to detect different signals that are hard to detect when using linear models or shallow sigmoid neural networks. Among them, the gene *GRM2* is the top pick. Although the biological mechanism of the association between these genes and AD needs further validation, it is worth pointing out that a recent study has shown that the metabotropic glutamate receptor 2 (mGluR2), a protein encoded by the gene *GRM2* plays a role in the pathogenesis of AD (Srivastava et al., 2020).

## Discussions and conclusion

In this paper, we have proposed a goodness-of-fit test based on ReLU neural networks. The proposed test can be used to detect the significance of a predictor. Once the network structures are suitably chosen, the test statistics have an asymptotically normal distribution, making it easy to implement in practice. Simulation results have demonstrated that the proposed method can detect nonlinear underlying signals, and real data analysis also showed the potential that ReLU neural networks may detect signals that are hard to identify from linear models or even shallow sigmoid neural networks.

On the other hand, although the theoretical framework of the GoF test was proposed in this paper, in practice, the performance of a deep ReLU neural network also depends on the optimization algorithm used and the hyperparameters (e.g., learning rate, number of epochs, etc.) selected. So, there is still a gap in how the DNN can be used to conduct statistical inference on detecting significant variables. This will be our future work. In addition, while we mainly focused on testing a single variable (such as a gene expression in the real data analysis) in this paper, it is worthwhile to also investigate the performance of our proposed method on a wider range of datasets to evaluate the performance of the GoF test when testing a set of variants in a genetic region, such as in a chromosome or in a pathway. In addition, various significant testing procedures based on neural networks nowadays and as a future work, we plan to conduct a comprehensive comparison on these methods.

## Data Availability

The datasets presented in this study can be found in online repositories. The names of the repository/repositories and accession number(s) can be found in the article/supplementary material.
